# Trends and correlations of infection rates and drug resistance of *Escherichia coli* among ICU and Non-ICU patients in Guizhou, China

**DOI:** 10.1371/journal.pone.0346023

**Published:** 2026-04-07

**Authors:** Zeling Dong, Xuejiao Mu, Wensheng Du, Zhengyuan An, Jialin Xiang, Anxin Yang, Qiongyao Li, Zuyi Chen

**Affiliations:** 1 Department of Laboratory Medicine, Affiliated Hospital of Zunyi Medical University, Zunyi, Guizhou, P.R. China; 2 Department of Medical Laboratory, People’s Hospital of Dejang, Dejang, Guizhou, China; 3 Information Division, Affiliated Hospital of Zunyi Medical University, Zunyi, Guizhou, China; Universidad San Francisco de Quito, ECUADOR

## Abstract

**Objective:**

*Escherichia coli* (*E. coli*) is a common opportunistic pathogen. The objective of this study was to analyze the characteristics, trends, and correlations *E. coli* infection rates and drug resistance patterns between intensive care unit (ICU) and non-ICU patients in Guizhou, China. These findings can provide valuable information for treating *E. coli* infections and enriching the theoretical framework for controlling bacterial infections and drug resistance.

**Methods:**

Samples were collected from both outpatients and inpatients. Identification of bacterial species, extended-spectrum β-lactamase (ESBL) testing, and drug susceptibility testing for 17 antibiotics were performed using the VITEK-2 Compact system. Trends were examined using correlation analyses with a two-tailed Pearson’s test in SPSS 26.0.

**Results:**

*E. coli* was the most frequently detected pathogen. Infection rates showed a decreasing trend, which positively correlated with declining ESBL-positive rates, comprehensive drug resistance rates (defined as the ratio of total resistant results to total tests across 17 antimicrobial agents), and multidrug resistance (MDR) rates. Furthermore, the trend in ICU detection rates was positively correlated with declines in comprehensive drug resistance and MDR rates.

**Conclusion:**

Declining infection and ICU detection rates of *E. coli* were associated with reduced drug resistance rates. Controlling infection and drug resistance rates in *E. coli* may synergistically improve clinical outcomes.

## 1.  Introduction

*Escherichia coli* (*E. coli*) is a most common opportunistic pathogen associated with clinical infections [1]. It primarily causes urinary tract infections and can lead to bloodstream infections, neonatal meningitis, pyelonephritis, cystitis, and prostatitis [2,3].

Antibiotics remain the most effective means of treating bacterial infections. However, bacteria can gradually develop resistance to antibiotics. Drug resistance represents a key mechanism through which pathogens adapt to the environment and sustain infections. Antibiotics kill sensitive bacteria but spare resistant bacteria, enabling resistant bacteria to gain a survival advantage and increase their proportion within the bacterial population, thereby enhancing their overall resistance [4]. Prolonged and frequent antibiotic use during infections can promote the emergence and development of drug resistance [5]. Bacterial drug resistance has emerged as a major challenge in clinical anti-infection therapy, and controlling resistant bacteria has long been a central focus of medical practice. Various guidelines and standards for antibiotic use have been established to curb the rise of antibiotic resistance [6]. Nevertheless, despite continued antibiotic use and the efforts of medical institutions to control bacterial resistance, the drug resistance profiles of bacteria may still change, potentially altering patterns of bacterial infection. A meta-analysis identified a positive correlation between the incidence of *Clostridium difficile* infection and drug resistance rates, whereas other reports showed that community-acquired *Staphylococcus aureus* infection was not significantly associated with drug resistance [7–9]. However, primary data on drug resistance trends and their relationship with pathogenicity remain scarce, warranting further investigation. Monitoring and analyzing the infection characteristics and drug resistance of *E. coli*, the most frequently isolated pathogen in clinical infections, is particularly crucial [10].

Notably, bacterial distribution and drug resistance vary across regions [11]. To date, no studies have reported the infection characteristics and resistance of *E. coli* in Guizhou Province. To guide risk group assessment and support informed medication decisions, this study analyzed the infection profiles and drug resistance characteristics of *E. coli* in Guizhou Province, including the extended-spectrum beta-lactamase (ESBL)-positive rate. Furthermore, this study explored the correlation between the changing trends in positive rates, resistance rates, and intensive care unit (ICU) detection rates of *E. coli* (ICU detection rates represent the percentage of all *E. coli* cases that came from the ICU). Controlling drug resistance could complement efforts in infection control, potentially providing new perspectives to advance understanding of bacterial infection, resistance prevention, and control.

## 2.  Materials and methods

This retrospective study was approved by the Ethics Committee of the Affiliated Hospital of Zunyi Medical University (Approval no. KLL-2020–008). As the study utilized routinely collected diagnostic data and all individual patient information was anonymized prior to analysis, the requirement for informed consent was waived. All methods were performed in accordance with the relevant guidelines and regulations stipulated by the Ethics Committee. A consecutive sampling method was adopted for this study, and patient data were retrieved via patient IDs. Inclusion criteria: All test results of patients who underwent bacterial culture and identification at the Affiliated Hospital of Zunyi Medical University from January 1, 2016 to December 31, 2022. Exclusion criteria: Duplicate test results from the same patient were excluded—for patients with multiple batches of samples, only the results of the first batch were included; if multiple samples were submitted in the first batch, only non-duplicate positive results were retained. Samples with missing ESBL or antibiotic susceptibility test results were excluded from the corresponding analyses, respectively.

### 2.1.  Sample collection

Samples were collected from patients at the Affiliated Hospital of Zunyi Medical University and included blood, cerebrospinal fluid, bone marrow, bile, drainage fluid, amniotic fluid, abscess secretions, cervical/vaginal secretions, sputum, throat swabs, urine, prostatic fluid, semen, bronchoalveolar lavage fluid, dialysate, and other clinical material from patients with suspected bacterial infections.

### 2.2.  Bacterial identification and antimicrobial susceptibility testing

Following standard culture procedures, bacterial identification, ESBL detection, and antimicrobial susceptibility testing were performed using the VITEK-2 Compact Automated Microbiology System (BioMérieux, France) using manufacturer-specified reagents. Drug susceptibility testing for the 17 antimicrobial agents or combinations was conducted in compliance with the annual Clinical and Laboratory Standards Institute guidelines for Antimicrobial Susceptibility Testing (M100-S26 to M100-S32) corresponding to each study year from 2016 to 2022.

### 2.3.  Data analysis

Data analysis was performed using Microsoft Excel 2021 for descriptive statistics, and SPSS 26 (IBM Corp., Armonk, NY, USA) for relative risk (RR), chi-square tests, and two-tailed Pearson correlation analyses. Age groups were stratified according to the WHO classification: children (0‒14 years), young adults (15‒47 years), middle-aged adults (48‒63 years), and older adults (≥64 years). Seasonal divisions followed Chinese meteorological standards (QX/T 152−2012 and GB/T 42074−2022): spring (March‒May), summer (June‒August), autumn (September‒November), and winter (December‒February).

The comprehensive drug resistance rate was calculated as the ratio of total resistance to total tests for all 17 antimicrobial agents. Multidrug-resistant organisms were defined as those resistant to ≥3 antimicrobial classes, in accordance with the Chinese Ministry of Health guidelines. ICU admission criteria were based on the Chinese national standards for ICU management.

### 2.4.  Sample size calculation

Sample size was determined based on the primary outcome measure: the correlation between *E. coli* positivity rates and comprehensive drug resistance rates. Statistical power analysis was performed using G*Power 3.1 with Pearson correlation analysis (two-tailed), α = 0.05 (type I error rate), power (1-β) = 0.90 (type II error rate), and an expected effect size (r) = 0.10. The minimum required sample size was calculated as 789 cases. This study included 9,247 *E. coli* strains with complete drug susceptibility data.

## 3.  Results

A total of 340,824 participants were included in this study, among whom 11,099 were positive for *E. coli*, representing the highest detection rate among all bacteria (16.773%, 11,099/63,786). The number of various samples and the distribution of *E. coli*-positive samples across each sample type are shown in Table 1. Owing to patient non-consent, ESBL testing was performed on 10,059 *E. coli* samples, and complete drug susceptibility testing for all 17 agents was conducted on 9,247 of these ESBL-tested samples. Detailed raw data are presented in S1–S5 Files.

**Table 1:** Counts and Positive Rates of Various Samples.

Sample type	*E. coli* positive samples	Total samples	Positive rate
Urine	4555	9622	47.34%
Whole blood	2153	10007	21.51%
Secretions	1832	15666	11.69%
Sputum	891	19300	4.62%
Cervical secretions	475	874	54.35%
Puncture fluid	312	1442	21.64%
Drainage fluid	296	1016	29.13%
Cavity effusion	260	1223	21.26%
Bile	119	401	29.68%
Cerebrospinal fluid	55	1047	5.25%
Bronchoalveolar lavage fluid	38	1033	3.68%
Other clinical material	32	260	12.31%
Tissue	28	214	13.08%
Catheter specimen	18	536	3.36%
Throat swabs	11	414	2.66%
Feces	8	576	1.39%
Semen	6	49	12.24%
Prostatic fluid	4	75	5.33%
Dialysate	2	3	66.67%
Gastric contents	2	3	66.67%
Gastric juice	1	3	33.33%
Amniotic fluid	1	3	33.33%
Breast milk	0	8	0.00%
Bone marrow	0	11	0.00%
Total	11099	63786	17.40%

Note: Numbers for “*E. coli* positive samples” and “Total samples” represent the count of *E. coli*-positive samples and the total number of samples for each type, respectively.

### 3.1.  *E. coli* infection characteristics

*E. coli* infection rates varied significantly according to sex, season, and age. Females had a higher RR than males (RR = 2.24, *p* < 0.001). The highest RRs were observed in summer (RR = 1.09, *p* = 0.005) and among middle-aged individuals (RR = 2.81, *p* < 0.001), as detailed in Table 2.

**Table 2:** Sex, season, and age characteristics of *Escherichia coli* infection.

Groups	Positive number	Negative number	RR	*p*	χ2
Sex				<0.001	1886.34
Female	6699	113286	2.24		
Male	4400	166391	1		
Season				0.005	12.99
Spring	2649	80394	1.02		
Summer	3171	89450	1.09		
Autumn	2809	83601	1.04		
Winter	2470	76280	1		
Age group				<0.001	1069.89
0-14	1130	76372	1		
15-47	3280	88107	2.46		
48-63	3537	82863	2.81		
≥64	3152	82383	2.53		

Note: The lowest positive rate was selected as comparison unit 1. RR represents the positive rate ratio.

### 3.2.  *E. coli* drug resistance characteristics

The overall drug resistance rate was significantly higher in males than in females (RR = 1.1, *p* < 0.001). Resistance rates peaked in summer (RR = 1.02, *p* = 0.027) and in middle-aged patients (RR = 1.1, *p* < 0.001). ICU patients exhibited significantly higher rates of multidrug resistance (MDR; RR = 1.31, *p* = 0.025) and comprehensive drug resistance (RR = 1.07, *p* = 0.003) than non-ICU patients, although ESBL-producing *E. coli* rates did not differ significantly between the groups (*p* = 0.117) (Table 3).

**Table 3:** Differences in Drug Resistance by Gender, Age, Season, and in the ICU.

Groups	Resistant	Non-resistant	RR	*p*	χ2
Gender				<0.001	32.89
Female	37686	56873	1		
Male	25876	36771	1.06		
Season				0.027	9.15
Spring	14995	22410	1.00		
Summer	17997	26030	1.02		
Autumn	16582	24210	1.02		
Winter	13988	20994	1		
Age group				<0.001	114.45
0-14	6003	10029	1		
15-47	18443	28054	1.06		
48-63	20936	29267	1.11		
≥64	18180	26294	1.09		
MDR				0.025	5.04
Detection rate in ICU	462	84	1.31		
Detection rate in non-ICU	7024	1677	1		
Comprehensive drug resistance				0.003	9.02
Detection rate in ICU	3887	5340	1.07		
Detection rate in non-ICU	59675	87512	1		
ESBL	Positive	Negative		0.117	2.46
Detection rate in ICU	393	181	1.05		
Detection rate in non-ICU	6190	3295	1		

Note: The lowest positive rate in each group was selected as the comparison unit 1. RR represents the positive rate ratio. MDR: multidrug-resistant, ESBL: extended-spectrum β-lactamase.

### 3.3.  Trends in infection, resistance, and correlations

**Fig 1:**
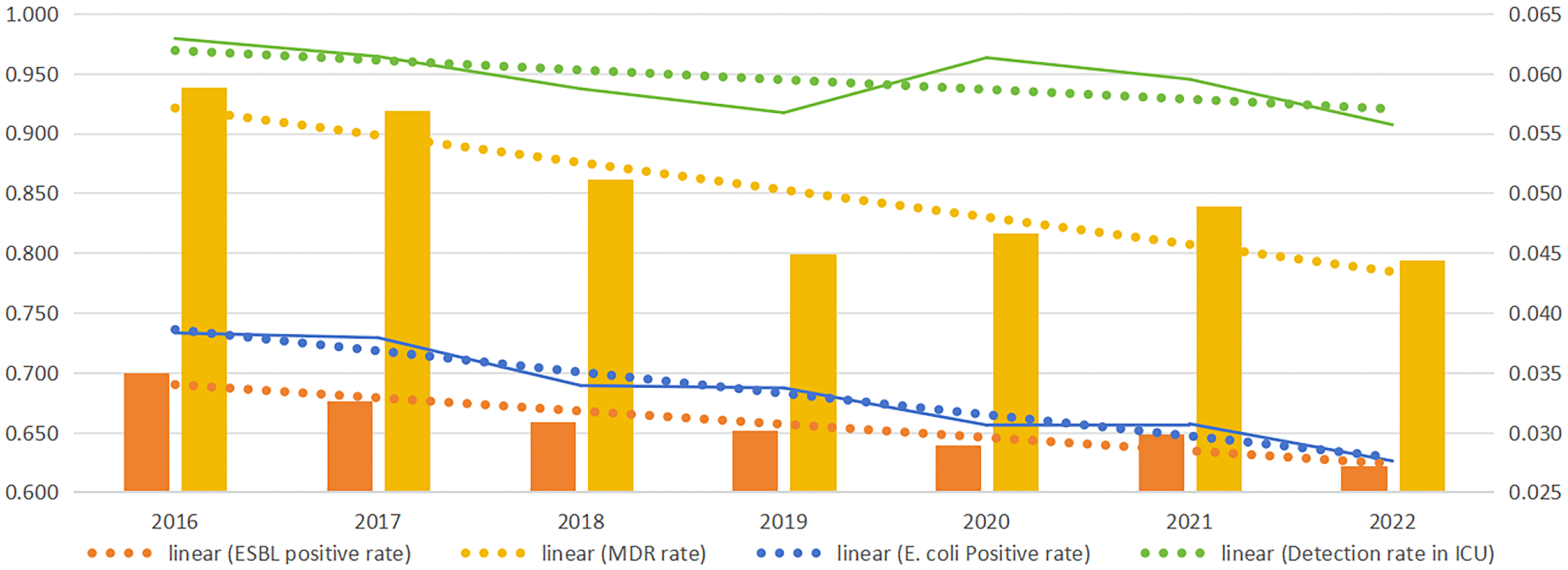
Linearity of change trends. Note: Solid lines and columns represent the rates. The dotted line represents the linear trend of the rates, and trend linearity was created using Microsoft Excel 2021..

From 2016 to 2022, significant decreasing trends were observed in *E. coli* positivity rates (*p* < 0.001), ESBL-producing *E. coli* rates (*p* = 0.02), comprehensive resistance rates (*p* < 0.001), and MDR rates (*p* < 0.001). Among *E. coli*-positive patients, the proportion of ICU cases decreased from 6.29% to 5.57%, although this decrease was not statistically significant (*p* = 0.922) (Table 4 and Fig 1).

**Table 4:** Trend of *E. coli* infection, ESBL positivity, MDR, and detection rate in ICU.

	*E. coli* Positive rate	ESBL positive rate	MDR rate	Detection rate in ICU
2016	991 (3.83%)	658 (70.00%)	686 (93.84%)	46 (6.29%)
2017	1674 (3.79%)	1059 (67.67%)	1092 (91.92%)	73 (6.14%)
2018	1856 (3.39%)	1105 (65.89%)	1253 (86.18%)	89 (5.87%)
2019	1700 (3.37%)	1005 (65.13%)	1120 (79.89%)	84 (5.67%)
2020	1461 (3.06%)	848 (63.90%)	1014 (81.64%)	81 (6.13%)
2021	1639 (3.07%)	913 (64.84%)	1123 (83.87%)	84 (5.95%)
2022	1778 (2.76%)	995 (62.23%)	1198 (79.44%)	89 (5.57%)
p for trend	<0.001	0.02	<0.001	0.922
χ2	135.339	21.201	161.755	0.988
conclusion	Significantly decreased	Significantly decreased	Significantly decreased	Decreased but not significantly

ESBL: extended-spectrum beta-lactamase. MDR: multidrug-resistant.

**Fig 2:**
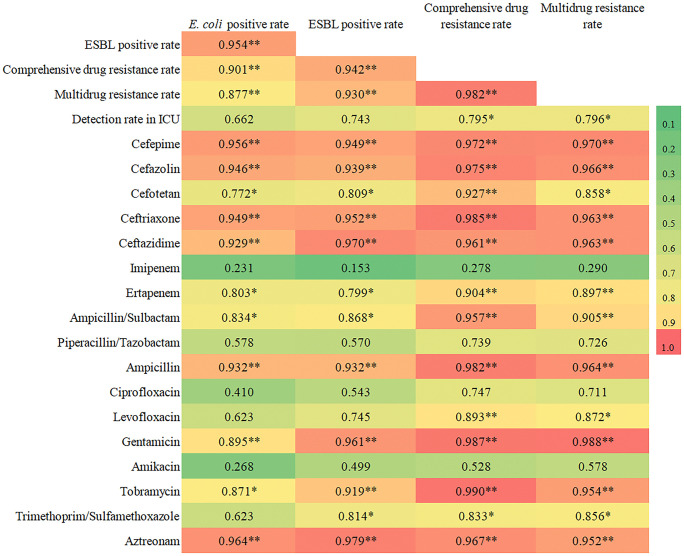
Correlation coefficient of rate trends. Note: The value is the correlation coefficient (r) corresponding to the change trends of two rates. The blank part represents the repeated corresponding relationship. **: Significant correlation at the *p* < 0.01 level (two-tailed). *: Significant correlation at the 0.01 < *p* < 0.05 level (two-tailed). ESBL: extended-spectrum beta-lactamase. MDR: multidrug-resistant. FEP: Cefepime, CFZ: Cefazolin, CTT: Cefotetan, CRO: Ceftriaxone, CAZ: Ceftazidime, IPM: Imipenem, ETP: Ertapenem, SAM: Ampicillin/Sulbactam, TZP: Piperacillin/Tazobactam, AMP: Ampicillin, CIP: Ciprofloxacin, LEV: Levofloxacin, GEN: Gentamicin, AMK: Amikacin, TOB: Tobramycin, SXT: Trimethoprim/Sulfamethoxazole, ATM: Aztreonam, Total: Comprehensive drug resistance rate.

Among the 17 agents tested, 16 showed significant reductions in resistance rates (*p* < 0.001), whereas amikacin showed irregular fluctuations (*p* = 0.171) (Table 5). Decreasing trends in comprehensive resistance and MDR rates were positively correlated with trends for 13 antimicrobial agents (Fig 2).

**Table 5:** Trend of 17 drug resistance rates.

Drug	2016 n = 731	2017 n = 1188	2018 n = 1517	2019 n = 1481	2020 n = 1321	2021 n = 1411	2022 n = 1598	*p* for trend
FEP	36.94%	36.87%	29.00%	24.38%	24.75%	24.81%	19.40%	<0.001
CFZ	100.00%	98.99%	83.12%	75.30%	72.07%	73.42%	69.40%	<0.001
CTT	5.47%	5.56%	2.31%	2.90%	2.95%	3.61%	2.63%	<0.001
CRO	95.90%	93.52%	76.20%	71.37%	67.60%	69.53%	64.27%	<0.001
CAZ	49.79%	44.11%	39.16%	33.69%	31.87%	32.96%	30.35%	<0.001
IPM	0.82%	2.69%	1.25%	1.22%	0.76%	2.41%	1.00%	<0.001
ETP	4.24%	5.56%	2.44%	1.69%	1.14%	3.12%	1.38%	<0.001
SAM	79.21%	78.70%	59.26%	60.43%	63.89%	65.06%	56.13%	<0.001
TZP	3.69%	5.30%	2.64%	2.36%	2.27%	4.04%	2.57%	<0.001
AMP	99.59%	99.75%	94.00%	92.37%	90.92%	92.63%	90.61%	<0.001
CIP	74.15%	71.66%	60.91%	58.88%	67.83%	67.47%	65.96%	<0.001
LEV	71.68%	67.85%	58.40%	54.83%	61.77%	61.94%	59.70%	<0.001
GEN	53.63%	49.66%	44.17%	39.77%	41.26%	42.38%	37.98%	<0.001
AMK	2.74%	1.94%	1.98%	1.35%	2.27%	2.55%	1.63%	0.171
TOB	25.99%	24.49%	15.23%	14.45%	15.14%	17.01%	12.58%	<0.001
SXT	68.26%	61.78%	59.79%	55.98%	57.08%	62.93%	57.38%	<0.001
ATM	67.99%	62.79%	53.92%	49.90%	46.18%	47.06%	42.62%	<0.001
Total	49.42%	47.72%	40.22%	37.71%	38.22%	39.58%	36.21%	<0.001

Note: FEP: Cefepime, CFZ: Cefazolin, CTT: Cefotetan, CRO: Ceftriaxone, CAZ: Ceftazidime, IPM: Imipenem, ETP: Ertapenem, SAM: Ampicillin/Sulbactam, TZP: Piperacillin/Tazobactam, AMP: Ampicillin, CIP: Ciprofloxacin, LEV: Levofloxacin, GEN: Gentamicin, AMK: Amikacin, TOB: Tobramycin, SXT: Trimethoprim/Sulfamethoxazole, ATM: Aztreonam, Total: Comprehensive drug resistance rate.

*E. coli* positivity was strongly correlated with trends in ESBL-producing *E. coli* (r = 0.95, *p* = 0.001), comprehensive resistance (r = 0.90, *p* = 0.006), and MDR (r = 0.88, *p* = 0.01). Trends in resistance to 11 drugs were positively correlated with *E. coli* positivity (Fig 2).

Comprehensive resistance (r = 0.94) and MDR trends (r = 0.93) were also positively correlated with ESBL-producing *E. coli* trends, with 12 drug resistance trends aligning with ESBL trends (Fig 2).

Trends in ICU detection rates showed no significant correlation with trends in *E. coli* positivity (*p* = 0.105) or ESBL-producing *E. coli* rates (*p* = 0.054), but were positively correlated with decreasing comprehensive resistance (r = 0.80) and MDR trends (r = 0.80) (Fig 2). Complete correlation details are provided in S1 File.

## 4.  Discussion

*E. coli* is the most frequently isolated opportunistic pathogen in clinical settings, making understanding its infection patterns and drug resistance crucial for infection control and antimicrobial stewardship [12–14]. Based on infection and drug resistance data on *E. coli* in Guizhou, China, from 2016 to 2022, this study analyzed the correlation among its positive rates, drug resistance characteristics, and changing trends in ICU and non-ICU detection rates. Overall, both positive and comprehensive resistance rates of these *E. coli* strains declined gradually. Notably, this study revealed a positive correlation between *E. coli* positivity rates and comprehensive drug resistance rates. Additionally, trends in ICU detection rates were positively correlated with trends in comprehensive drug resistance and MDR rates. These findings offer new insights that may advance the theoretical understanding of pathogen infection and the control of resistance.

The sex distribution of *E. coli* infections showed a higher prevalence in females than in males, consistent with previous reports [15,16]. This is likely because *E. coli* is part of the intestinal flora and the female urethra is anatomically closer to the rectum, which increases the susceptibility to secondary infections [15,16]. Nevertheless, the drug resistance rate was slightly higher in males than in females. The contrasting patterns between infection prevalence and resistance rates, similar to those reported in previous studies, suggest that factors beyond drug resistance survival capabilities may influence sex-specific differences. For example, because *E. coli* is more common in urinary tract infections, a longer male urethra may prolong infection and treatment duration, potentially leading to higher drug resistance [16].

Summer was identified as the peak season for *E. coli* infections, followed by autumn, spring, and winter, consistent with previous reports on *E. coli* bacteremia [16,17]. Similarly, the seasonal peak of drug resistance also occurred in summer, maintaining consistency with the infection rates. The peak age for *E. coli* infections was middle adulthood, followed by older adults, young adults, and children. This pattern differs from the age-related increase in *E. coli* bacteremia rates, indicating site-specific infection patterns [18].

The notable differences in drug resistance between ICU and non-ICU patients suggest that bacterial resistance, especially MDR, may lead to more severe infections, as reported previously [19]. Furthermore, bacterial resistance is associated with a poor prognosis [20]. Timely selection of appropriate antimicrobial agents is critical for treating bacterial infections [21]. However, primary healthcare institutions, major participants in healthcare, often lack the capability to test for bacterial resistance and rely on guidelines and experience in antibiotic use [22].

In this study, we analyzed the drug resistance of 11,099 *E. coli* strains. Resistance rates for most antimicrobial agents showed sustained decline, consistent with a recent report from Zhejiang, China [23]. Notably, resistance rates for some drugs (e.g., ampicillin, 90.61%; cefazolin, 69.40%) remained persistently high, limiting their clinical utility. Therefore, empirical therapy for *E. coli* infections should prioritize carbapenems, beta-lactamase inhibitor combinations (e.g., piperacillin/tazobactam), ceftazidime, and amikacin (with caution for nephrotoxicity).

ESBLs are enzymes that hydrolyze the β-lactam ring, inactivating antibiotics. ESBL-producing bacteria develop resistance to most β-lactams, including penicillins, cephalosporins, and aztreonam [24]. In this study, the proportion of ESBL-producing *E. coli* decreased considerably over the seven-year study period. However, by 2022, 62.23% of strains were ESBL-positive, a rate higher than those reported in the United States (1.8%‒8%), Europe (3.3%‒23.6%), and South Asia (33.2%), but comparable to rates in Henan and Zhejiang provinces [23,25,26]. The decline in ESBL-positive strains was associated with reduced resistance to cephalosporins, penicillins, and aztreonam. Decreasing resistance rates to non-ESBL-related drugs (e.g., meropenem) may reflect indirect associations. Although ESBL-producing pathogens have been linked to severe symptoms and poor prognosis [27], this study found no significant difference in ESBL rates between ICU and non-ICU infections (*p* = 0.117), and ICU detection rates were not correlated with ESBL trends. This lack of significant difference may reflect regional or population-specific characteristics in Guizhou, where ESBL-producing *E. coli* has become widely disseminated across both ICU and non-ICU settings.

Limitations: Despite the large overall sample, the relatively small number of ICU cases with *E. coli* infection may have limited the ability to detect significant trends in ICU detection rates, and may also explain the lack of significant difference observed in ESBL rates between ICU and non-ICU patients.

## Supporting information

S1 File Detailed results of correlation analysis. (XLS)

S2 File Gender, Age and Testing Time of Patients with *E. coli* Positivity. (XLSX)

S3 File Supplementary material 3 Results of ESBL Detection. (XLSX)

S4 File Susceptibility Test Results of 17 Antibiotics or Antibiotic Combinations. (XLSX)

S5 File Count of *E. coli* Samples From ICU. (XLSX)

## Abbreviations

E. coli: Escherichia coli
RR: relative risk
ESBL: extended-spectrum beta-lactamase
MDR: multidrug-resistant
ICU: intensive care unit
